# Case of Dry Gangrene, Cured by Amputation

**Published:** 1839-03-16

**Authors:** William Zollickoffer

**Affiliations:** Middleburg, Carroll co. (Md.)


*Case of Dry Gangrene, cured by Amputation.* By WILLIAM ZOLLICKOFFER, M. D.

The particulars of the following case that came under my immediate notice, thirteen years, last fall, I find recorded in my note book.

I was invited to a consultation with the late distinguished Doctor Annan, of Emmittsburg, Doctors W. B. Hebbard and John Swope, in the case of Mr. V——. Dr. Swope, who had been attending him, stated that some months previous Mr. V. had sustained considerable injury of the knee, by his horse running him, in a fright, against a tree, from which he had recovered at least two months before any symptoms of *dry mortification* were discovered. The disease, he remarked, made its first appearance on the toes: when I witnessed it, it extended an inch above the ankle. At this time his general health was very much impaired, having laboured under hectic symptoms for about three weeks. The only remedy that presented itself, worthy of having recourse to, in order to save the life of Mr. V., we concluded would be found in amputation, and this was of doubtful efficacy, inasmuch as his system seemed to suffer much from the diseased condition of the affected parts. As to the point at which it should be performed, a difference of opinion existed between the three gentlemen and myself; all of whom insisted on the operation being made below the knee, in which I had to yield in consequence of being overruled. Thus situated, it fell to my lot to use the knife; previous, however, to operating, I gave it as my decided sentiment, from the disposition of the leg to run rapidly into *dry gangrene*, that a healthy stump could not be procured. The result terminated as I had predicted; the soft parts exhibited a dark livid aspect, in conjunction with the strongest evidences of an *entire* obliteration, produced by the injury of his knee, no doubt, of the vessels by which the leg and foot were usually supplied with blood. A second operation was now considered *essentially* necessary; and the impaired health of Mr. V. rendered it advisable that it should be performed without delay. Of this circumstance he was made acquainted. He stated he could "stand having his thigh cut off, as the other operation gave him very little pain." A potion of brandy and water was given him; in twenty minutes after, at the *special* request of my professional friends, I performed a second operation, about six inches above the knee. The stump showed a healthy condition. The operation being successful, the parts healed with the usual freedom; his hectic symptoms gradually, gave way, and in a few months Mr. V. was seen hopping about upon his crutches in fine health and spirits. When the operation was performed he was about twenty-one years of age. He has married since—at this time has three children, and has enjoyed better health than it was his lot to share before he was attacked by the diseased action, which rendered it necessary to amputate his limb. He is of a *strumous diathesis*.

*Middleburg, Carroll co.* (*Md.*) March 9, 1839.

